# Inhibitors of endosomal acidification suppress SARS-CoV-2 replication and relieve viral pneumonia in hACE2 transgenic mice

**DOI:** 10.1186/s12985-021-01515-1

**Published:** 2021-02-27

**Authors:** Chao Shang, Xinyu Zhuang, He Zhang, Yiquan Li, Yilong Zhu, Jing Lu, Chenchen Ge, Jianan Cong, Tingyu Li, Mingyao Tian, Ningyi Jin, Xiao Li

**Affiliations:** 1 Institute of Military Veterinary Medicine, Academy of Military Medical Sciences, Changchun, 130122 People’s Republic of China; 2 Academician Workstation of Jilin Province, Changchun University of Chinese Medicine, Changchun, 130117 People’s Republic of China; 3 Agricultural College, Yanbian University, Yanji, 133002 People’s Republic of China; 4 Jiangsu Co-Innovation Center for Prevention and Control of Important Animal Infectious Diseases and Zoonoses, Yangzhou, 225009 People’s Republic of China

**Keywords:** Endosomal acidification, SARS-CoV-2, Chloroquine, Bafilomycin A1

## Abstract

**Background:**

Coronavirus disease 2019 (COVID-19) is caused by SARS-CoV-2 and broke out as a global pandemic in late 2019. The acidic pH environment of endosomes is believed to be essential for SARS-CoV-2 to be able to enter cells and begin replication. However, the clinical use of endosomal acidification inhibitors, typically chloroquine, has been controversial with this respect.

**Methods:**

In this study, RT-qPCR method was used to detect the SARS-CoV-2N gene to evaluate viral replication. The CCK-8 assay was also used to evaluate the cytotoxic effect of SARS-CoV-2. In situ hybridization was used to examine the distribution of the SARS-CoV-2 gene in lung tissues. Hematoxylin and eosin staining was also used to evaluate virus-associated pathological changes in lung tissues.

**Results:**

In this study, analysis showed that endosomal acidification inhibitors, including chloroquine, bafilomycin A1 and NH_4_CL, significantly reduced the viral yields of SARS-CoV-2 in Vero E6, Huh-7 and 293T-ACE2 cells. Chloroquine and bafilomycin A1 also improved the viability and proliferation of Vero E6 cells after SARS-CoV-2 infection. Moreover, in the hACE2 transgenic mice model of SARS-CoV-2 infection, chloroquine and bafilomycin A1 reduced viral replication in lung tissues and alleviated viral pneumonia with reduced inflammatory exudation and infiltration in peribronchiolar and perivascular tissues, as well as improved structures of alveolar septum and pulmonary alveoli.

**Conclusions:**

Our research investigated the antiviral effects of endosomal acidification inhibitors against SARS-CoV-2 in several infection models and provides an experimental basis for further mechanistic studies and drug development.

## Background

Coronavirus disease 2019 (COVID-19) emerged in late 2019 and spread rapidly to become a global pandemic, posing significant threat to human health. This pathogen was officially named by World Health Organization (WHO) as severe acute respiratory syndrome coronavirus 2 (SARS-CoV-2). SARS-CoV-2 is a positive-sense and single-strand RNA virus and classified in the *Betacoronavirus* genera of the Coronaviridae family that is known to infect mammals [1, 2].

SARS-CoV-2 binds to the human angiotensin-converting enzyme 2 (hACE2) protein in human cells using spike protein and induces fusion between viral and cellular membranes [3, 4]. The virus is thought to be enclosed into vesicles which are then transported through the endosomal pathway. In order to begin viral replication, there are two essential requirements: endosomal acidification and the pH-dependent cleavage of viral glycoprotein segments by endosomal proteases. Without these processes, the virus cannot enter cells and replication is therefore abolished [5]. Therefore, agents that target endosomal acidification may represent potential antiviral approaches in the fight against SARS-CoV-2 [6].

Lysosomotropic agents, such as chloroquine and hydroxychloroquine, are effective therapeutics for the treatment of malaria and have been used in the treatment of COVID-19 [7]. It has been reported that chloroquine increases the pH of endosomes and therefore suppresses viral replication that depends on low pH for cell entry [8]. In Vero E6 cells, chloroquine has been shown to inhibit SARS-CoV-2 infection, thus providing a rational for the treatment of COVID-19 [9, 10]. However, research studies using calu-3 cells lines that are derived from human lung adenocarcinoma cells and cynomolgus macaque models have been shown to negate the therapeutic effects of chloroquine and hydroxychloroquine [11, 12]. Consequently, the use of chloroquine for the treatment of COVID-19 is the subject of significant debate. Therefore, different cell and animal models of SARS-CoV-2 infection are now needed to evaluate the true validity of chloroquine and other inhibitors of endosomal acidification, including bafilomycin A1 and NH_4_CL.

hACE2 plays a vital role in the cell entry process of SARS-CoV and SARS-CoV-2 [13–15]. A recent study reported that hACE2 transgenic mice are an applicable animal model for SARS-CoV-2 infection with increased viral load over time. In addition, in certain lung lobes of the infected mice, gross lesions with focal to multifocal dark red discoloration and infiltration of inflammatory cells into bronchiolar epithelial and alveolar interstitium were also observed [16].

As a sensitive cell model in addition to Vero E6 cells, 293T-hACE2 cells that express hACE2 in 293T cells can be used for SARS-CoV-2 infection and investigation of the cell entry mechanisms by pseudovirus [17]. Moreover, it was reported that Huh-7 cells were used for SARS-CoV-2 isolation and as a cell model for the evaluation of anti-SARS-CoV-2 activity by Lianhuaqingwen [18, 19].

In the present study, we used Vero E6, Huh-7 and 293T-hACE2 cells, along with hACE2 transgenic mice, as models for SARS-CoV-2 infection. We used these models to investigate the actions of chloroquine, bafilomycin A1 and NH_4_CL, on SARS-CoV-2 replication and virus-associated damage.

## Methods

### Chemicals, virus and cell lines

Chloroquine and NH_4_CL were purchased from Sigma-Aldrich (St. Louis., MO, USA). Bafilomycin A1 was purchased from MedChemExpress (New Jersey, USA). The SARS-CoV-2 strain BetaCoV/wuhan/AMMS01/2020 was originally isolated by CFQ’s laboratory at the Academy of Military Medical Sciences [20]. African green monkey kidney (Vero E6) cells were previously preserved in our laboratory. HEK293T-hACE2 stable cells were kindly provided by Zhong Ji Dang Kang Biotechnology Co. (Beijing, China). Human hepatoma cells (Huh-7) were purchased from the Committee on Type Culture Collection of Chinese Academy of Sciences (Shanghai, China). All cells were cultured in Dulbecco’s modified Eagle medium (DMEM) supplemented with 10% fetal bovine serum (FBS), 50 U ml^−1^ of penicillin and 50 µg ml^−1^ of streptomycin at 37℃ with 5% CO_2_. All experiments with live SARS-CoV-2 were conducted in a Biosafety Level 3 laboratory in the Institute of Military Veterinary Medicine, Academy of Military Medical Sciences.

### Cell viability assays

Cell viability was assayed by the CCK-8 regent (Dojindo Molecular Technologies, Rockville, USA) in accordance with the manufacturer’s instructions. In brief, Vero E6 cells (7 × 10^3^ cells/well) were transferred into a 96-well plate. After incubation for 24 h, treatments with viruses or drugs were applied. Chloroquine or bafilomycin A1 was given 2 h prior to virus treatment, the CCK-8 reagent was added (10 μl per well) after the indicated incubation times, and 450 nm OD values were determined with a multifunction microplate reader after 2 h of incubation.

### Crystal violet staining

Cell proliferation ability was examined as previously described using crystal violet staining [21]. In brief, Vero E6 cells (2 × 10^5^ cells/well) were transferred into 6-well plates and incubated for 24 h. Prior to virus administration, Vero E6 cells were pre-treated with chloroquine or bafilomycin A1 for 2 h. After incubation for 48 h, cells were then stained with crystal violet and fixed with 4% paraformaldehyde for 1 h. Cells were then analyzed by microscopy and representative images were captured with a digital camera.

### Animal experiments

Female hACE2 transgenic mice, aged 6 weeks, were in a C57/B6 background and obtained from the National Institute for Food and Drug Control (Beijing, China). Transgenic mice expressing hACE2 receptor are driven by the mice ACE2 promoter as described previously [16]. All animals were fed under conditions of controlled lighting (12 h light/dark), temperature (23 ± 1 °C), and humidity (45%). Mice were randomly distributed into experimental groups (n = 6) and the following experiments were conducted in a Biosafety Level 3 laboratory. Chloroquine (60 mg kg^−1^) or bafilomycin A1 (0.1 mg kg^−1^) was given intraperitoneally 2 h prior to SARS-CoV-2 injection and once daily for a further 5 days. Each mouse was intratracheally inoculated with 6.7 × 10^3^ PFU of SARS-CoV-2 in 30 μl of PBS under anesthesia by intraperitoneal barbiturates. On day 5, mice were sacrificed by cervical dislocation and the primary organs were collected for RNA extraction, in situ hybridization and histopathological examination. All experiments were performed in accordance with the National Institute of Health Guide for the Care and Use of Laboratory Animals.

### RNA extraction and qPCR

The viral RNA in cell supernatants or tissue homogenates was extracted by the QIAamp Viral RNA Kit (Qiagen, Hilden, Germany). Virus copies were then detected by RT-qPCR methods with the HiScript II One Step qRT-PCR SYBR Green Kit (Vazyme Biotech, Nanjing, China). The protocol for qRT-PCR was as follows: 50 °C for 15 min, 95 °C for 30 s, followed by 45 cycles at 95 °C for 10 s and 63 °C for 35 s. The primers used to detect the SARS-CoV-2N gene are as follows: Forward: GGG GAA CTT CTC CTG CTA GAA T; Reverse: CAG ACA TTT TGC TCT CAA GCTG. The PCR products were then examined using an ABI 7500 real time PCR system (Applied Biosystems, CA, USA).

### RNA in situ hybridization assay

Lung tissues were fixed in 4% paraformaldehyde solution containing 0.1% DEPC. Tissues were then dehydrated by a gradient of ethanol concentrations, embedded in paraffin wax, and cut into thin sections. The sequence of the probe used for RNA hybridization is as follows: 5´-DIG-ACTACAGCCATAACCTTTCCACATACCGCAGAC-DIG-3´. The DIG label was detected by an anti-DIG-HRP. After incubation with 3,3′-diaminobenzidine (DAB), the images were captured by light microscopy and the integrated optical density was analyzed by Image pro plus 6.0.

### Histopathological examination

Lung tissues were fixed in 4% paraformaldehyde solution and paraffin-embedded sections were prepared. Hematoxylin and eosin (H&E) staining was then used to identify pathological changes in the lung tissues. Images were then observed and captured by light microscopy.

### Statistical analysis

All data were analyzed by GraphPad Prism, Version 8.0. Data were analyzed with the t-test or by analysis of variance (ANOVA) followed by a two-tailed t-test and expressed as means ± SEM. *p* values < 0.05 were considered to be statistically significant.

## Results

### Inhibitors of endosomal acidification improved the viability of Vero E6 cells after SARS-CoV-2 infection

**Fig. 1:**
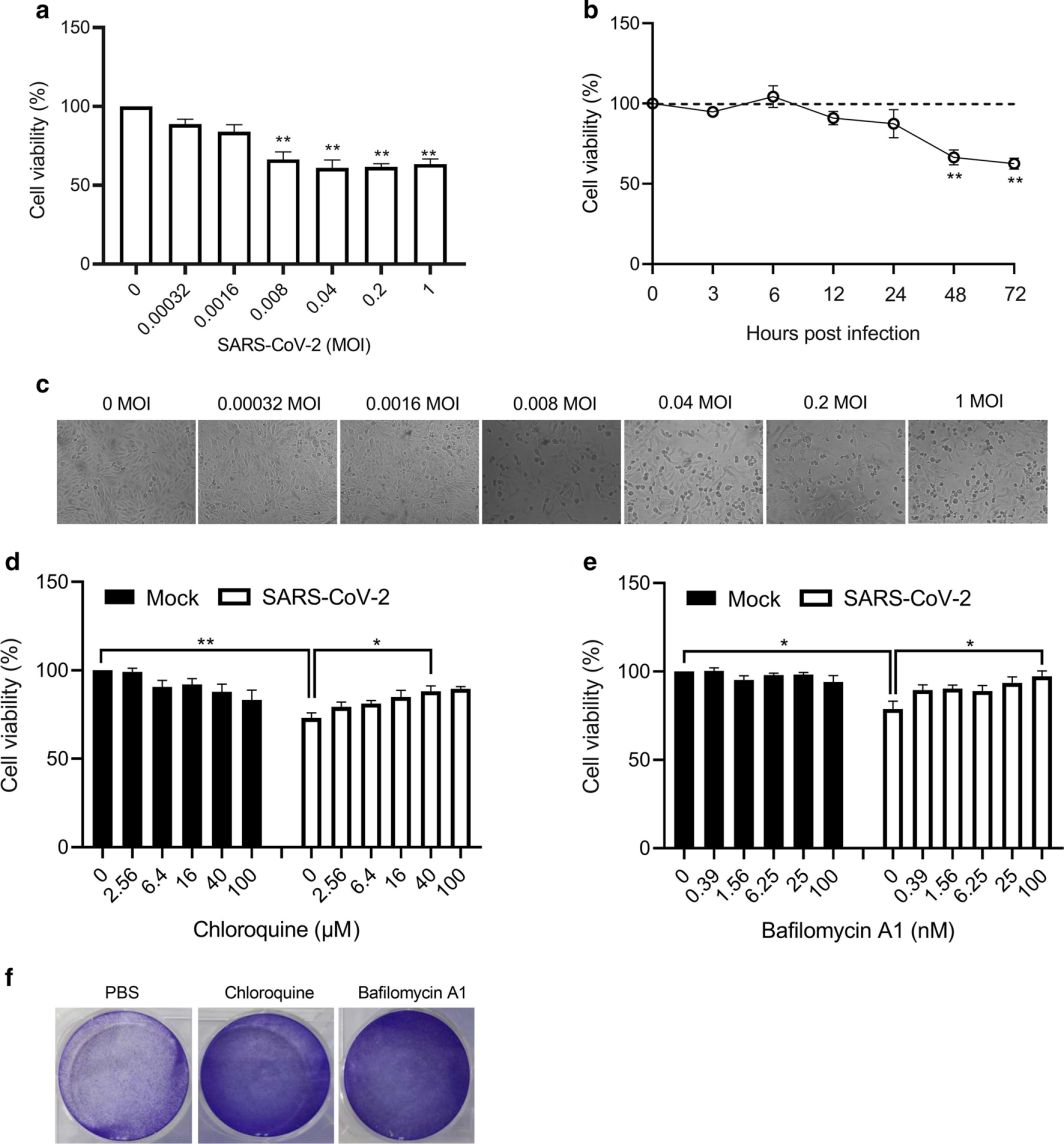
Inhibitors of endosomal acidification improved the viability of Vero E6 cells after SARS-CoV-2 infection. **a** Viability of Vero E6 cells infected with different doses of SARS-CoV-2 for 48 h. **b** Cell viability after SARS-CoV-2 infection (0.008 MOI) at different time points. **c** Images of Vero E6 cells challenged with 0.008 MOI of SARS-CoV-2 for 48 h. **d**, **e** Cell viability of Vero E6 cells treated with chloroquine or bafilomycin after SARS-CoV-2 infection (0.008 MOI) for 48 h. **f** Colony formation assays of Vero E6 cells treated with chloroquine (40 μM) or bafilomycin A1 (100 nM) after SARS-CoV-2 infection (0.008 MOI) for 48 h. Three experiments were performed (n = 6 each group). *p < 0.05, **p < 0.01

The infection dose and timing of SARS-CoV-2 infection were determined in Vero E6 cells using CCK-8 assays. Infection with 0.008–1 MOI of SARS-CoV-2 for 48 h significantly reduced cell viability in Vero E6 cells (Fig. 1a, b) and induced cytopathic effects (Fig. 1c). Chloroquine (40 μM), a lysosomotropic drug that rapidly crosses cell membranes and internalizes into endosomes and results in an increased pH, improved the viability of Vero E6 cells after SARS-CoV-2 infection at 0.008 MOI (Fig. 1d). Similarly, bafilomycin A1 (100 nM), an inhibitor of H^+^-ATPases that can acidify endosomes, also showed antiviral effects against SARS-CoV-2 (Fig. 1e). Correspondingly, colony assays showed that chloroquine and bafilomycin A1 improved cell proliferation in Vero E6 cells infected with SARS-CoV-2 (Fig. 1f).

### Inhibitors of endosomal acidification suppressed SARS-CoV-2 replication in vitro

**Fig. 2:**
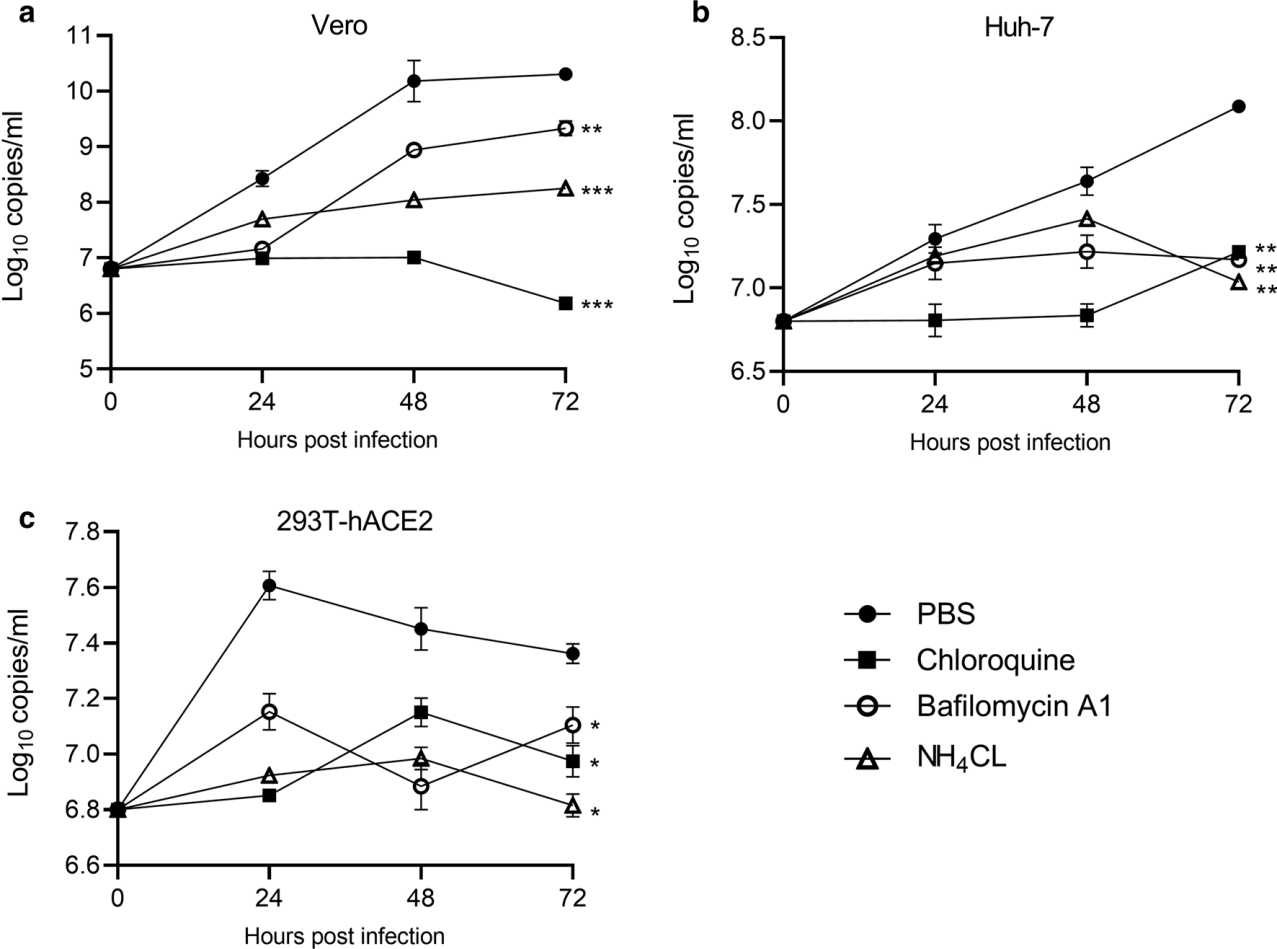
Inhibitors of endosomal acidification suppressed SARS-CoV-2 replication in vitro. Cells were infected with SARS-CoV-2 (0.008 MOI) and cell supernatant was harvested on indicated time points post infection. SARS-CoV-2 (0.008 MOI) was diluted in cell supernatant and harvested immediately as samples of 0 h post infection. Viral genome copies in Vero E6 cells (**a**), Huh-7 (**b**), and 293T-ACE2 cells (**c**) treated with PBS, chloroquine (40 μM), bafilomycin A1 (100 nM) or NH_4_CL (12.5 mM). Three experiments were performed (n = 3 each group). *p < 0.05, **p < 0.01, ***p < 0.001

To further investigate the antiviral effects against SARS-CoV-2 replication in different cell lines, we used human liver cells (Huh-7) and human kidney cells (293T-hACE2) in addition to Vero E6 cells. RT-qPCR results showed that chloroquine (40 μM), bafilomycin A1 (100 nM) and NH_4_CL (12.5 mM), suppressed the replication of SARS-CoV-2 in all cell types (Fig. 2a–c).

### Inhibitors of endosomal acidification suppressed SARS-CoV-2 replication hACE2 transgenic mice

**Fig. 3:**
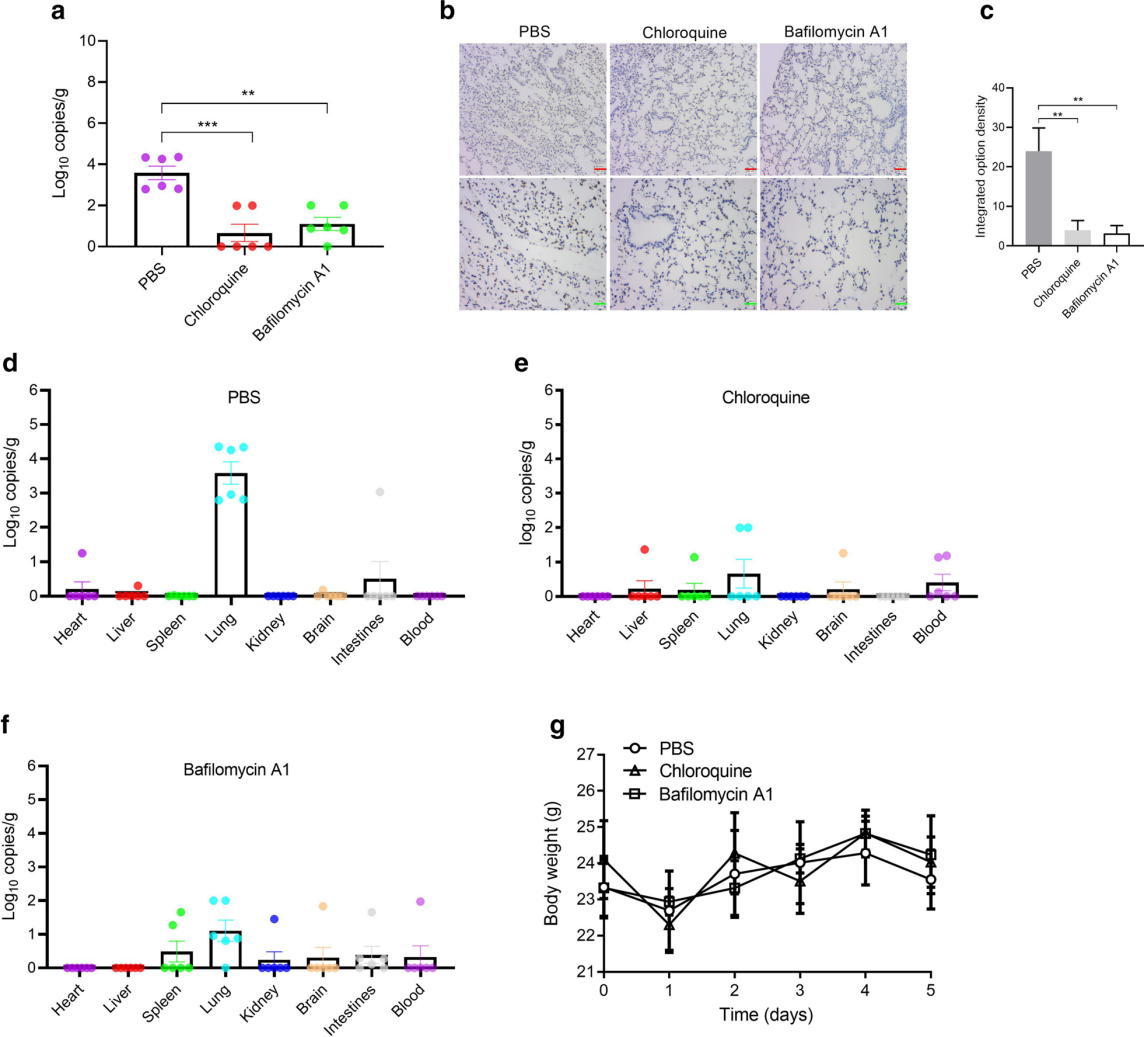
Inhibitors of endosomal acidification suppressed SARS-CoV-2 replication in hACE2 transgenic mice. hACE2 transgenic mice were inoculated with 6.7 × 10^3^ PFU of SARS-CoV-2 intratracheally in 30 μl of PBS. PBS, chloroquine (60 mg kg^−1^ once daily), or bafilomycin A1 (0.1 mg kg^−1^ once daily), were then given intraperitoneally for 5 days. Organ tissues were collected on day 5. **a** Viral genome copies in lung tissues. **b**, **c** In situ hybridization for SARS-CoV-2 RNA in lung tissues. **d**–**f** Viral genome copies in primary organs. **g** Body weight changes. Three experiments were performed (n = 6 each group). **p < 0.01, ***p < 0.001. Green bar = 25 μm. Red bar = 50 μm

Next, we used hACE2 transgenic mice to establish an animal model of SARS-CoV-2 infection. Figure 3a shows that chloroquine (60 mg kg^−1^) and bafilomycin A1 (0.1 mg kg^−1^) markedly reduced virus yields in lung tissues, suggesting that viral replication had been suppressed by chloroquine and bafilomycin A1. In addition, RNA in situ hybridization also showed reduced hybridization signals in the lung tissues of drug-treated mice (Fig. 3b, c). Figure 3d shows that SARS-CoV-2 predominantly infected the lung tissues and was scarcely detected in other primary organs in PBS-treated mice. SARS-CoV-2 were barely detected in primary organs of mice treated with chloroquine or bafilomycin A1 (Fig. 3e, f). Chloroquine and bafilomycin A1 treatment did not induce any significant changes in the body weight of hACE2 transgenic mice (Fig. 3g).

### Inhibitors of endosomal acidification alleviated viral pneumonia in hACE2 transgenic mice

**Fig. 4:**
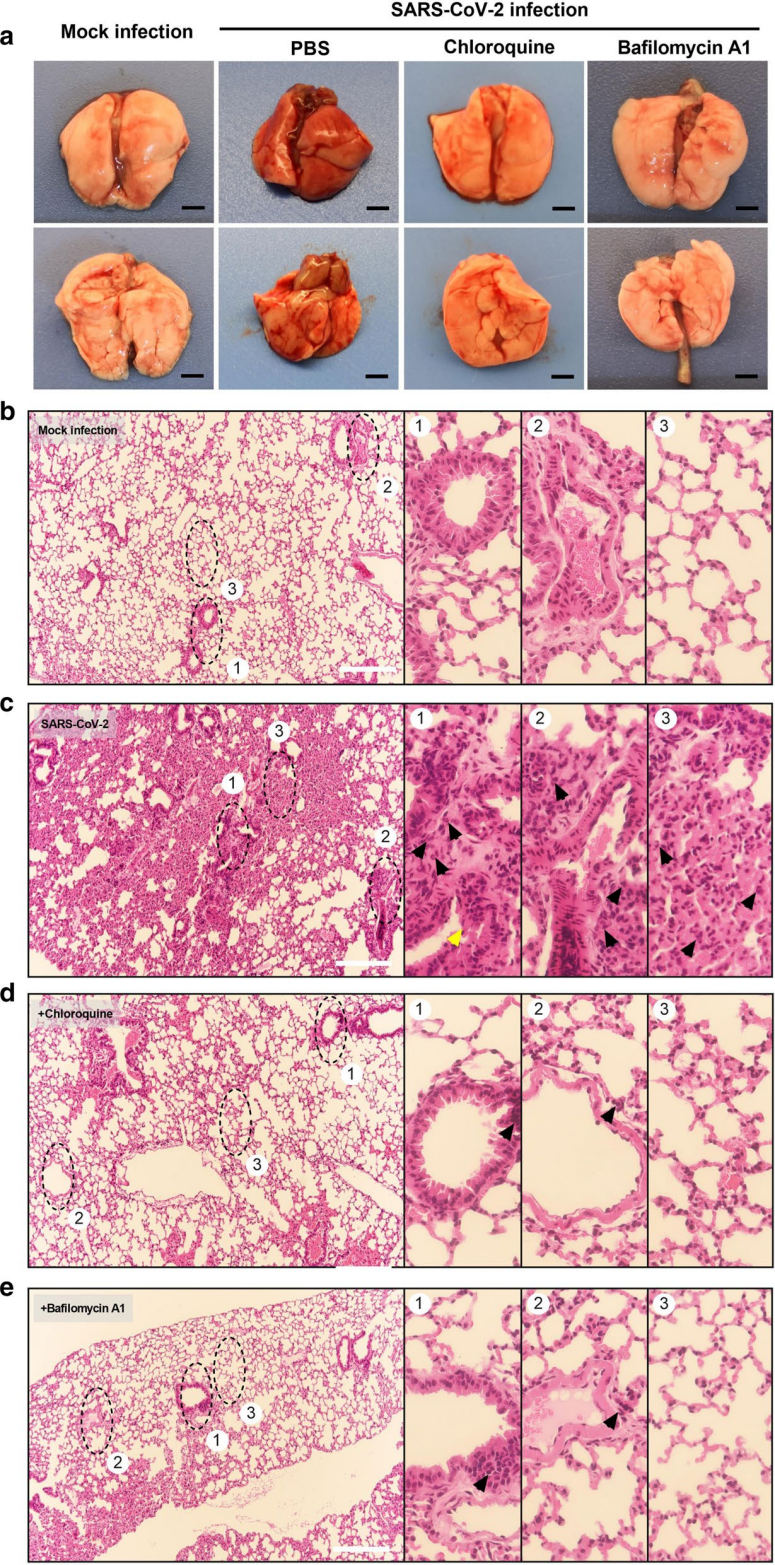
Inhibitors of endosomal acidification alleviated viral pneumonia in hACE2 transgenic mice. **a** Images of gross pathology of lungs from hACE2 transgenic mice subjected to indicated treatments. **b**, **c** Representative images for H&E staining of histopathological changes in lung tissues from Mock-infected (**b**) and SARS-CoV-2-challenged hACE2 transgenic mice (**c**). The numbered panels display magnified images of indicated black circles in the left. SARS-CoV-2 induced histopathological changes as follows: (1) bronchiolar epithelium cell death (yellow arrow) and peribronchiolar infiltration of segmented granulocytes or lymphocytes (black arrow). (2) Inflammatory exudation and infiltration in perivascular tissues (black arrow). (3) Collapse of alveolar structure and widened alveolar septum with inflammatory cell infiltration (black arrow). **d**, **e** Representative H&E staining images of lung tissues from SARS-CoV-2-challenged hACE2 transgenic mice that are subjected to chloroquine (**d**) and bafilomycin A1 (**e**) treatments. Reduced inflammatory exudation and infiltration in peribronchiolar (1) and perivascular (2) tissues, as well as improved structures of alveolar septum and pulmonary alveoli (3), were observed in chloroquine and bafilomycin A1 treatment groups. Representative images from 6 mice are shown. Black bar = 2 mm. White bar = 200 μm

Lung tissues from hACE2 transgenic mice were isolated 5 days after mock or SARS-CoV-2 infection. Gross pathology showed massive red lesions and discoloration in multiple lung lobes of SARS-CoV-2 challenged mice (Fig. 4a). The lung lobes become swollen and H&E staining showed severe pneumonia pathological changes as compared to mock infected groups with destruction of pulmonary alveolar structure and diffuse infiltration of inflammatory cells in alveolar septum (Fig. 4b, c). In addition, bronchiolar endothelial cell death and inflammatory segmented granulocytes infiltration in peribronchiolar and perivascular tissues were also observed (Fig. 4c). Lung tissues from chloroquine and bafilomycin A1 treatment groups showed less lesions in the surface (Fig. 4a) and microscopically alleviated inflammatory cell infiltration and improved structure of pulmonary alveoli (Fig. 4d, e).

## Discussion

Previous studies have demonstrated the importance of endosomal acidification for the activation of endosomal proteases and the infection of a range of viruses, including Marburg, Ebola, HIV, Dengue, and Chikungunya [22, 23]. Chloroquine and hydroxychloroquine have been reported to be capable of increasing acidic pH in the Golgi apparatus and inducing the dysfunction of various enzymes, including glycosylating enzyme which is known to suppress the glycosylation of SARS-CoV and reduce viral replication [8]. Thus far, the in vitro studies associated with the anti-viral efficacy of chloroquine against SARS-CoV-2 have been mainly performed in Vero E6 cells [9, 10, 24]. In this cell line, the EC50s for chloroquine were determined to be 23.90 μM at 24 h and 5.47 μM at 48 h [24]. Another study reported that the EC50s for chloroquine were 2.71, 3.81, 7.14, and 7.36 μM at different infection doses (0.01, 0.02, 0.2, and 0.8 MOI) [10]. Consistent with previous studies, the current study also demonstrated the antiviral effects of chloroquine (40 μM) against SARS-CoV-2 (0.008 MOI) infection in Vero E6 cells. Treatment resulted in increased cell viability and the inhibition of SAR-CoV-2 replication.

However, conflicting results in human cell lines, including Calu-3 cells (human lung adenocarcinoma cells) have cast doubt over the antiviral effects of chloroquine [11]. Therefore, in the present study, we used two human cell lines that were previously used for SARS-CoV infection: including Huh-7 (human heptocarcinoma cells) and 293T-hACE2 (human kidney cells) and showed that chloroquine (40 μM) markedly reduced viral replication. We also used the hACE2 transgenic mice model for SARS-CoV-2 infection, and demonstrated that chloroquine inhibited viral yields in lung tissues and alleviated pneumonia-associated pathological changes, thus indicating that chloroquine exhibited anti-SARS-CoV-2 effects. However, recent studies in cynomolgus macaques have demonstrated that hydroxychloroquine was not able to inhibit SARS-CoV-2 infection [12]. It is likely that differences between animal models may contribute to such conflicting results and further studies are now needed to fully elucidate the anti-SARS-CoV-2 effects of these drugs.

In addition to chloroquine, bafilomycin A1, and NH_4_CL, are also known to inhibit endosomal acidification [25]. Our current research showed that bafilomycin A1 and NH_4_CL significantly reduced the viral yields of SARS-CoV-2 in Vero E6, Huh-7 and 293T-hACE2 cells. Moreover, similar to chloroquine, bafilomycin A1 improved cell viability in virus-infected Vero E6 cells and protected hACE2 transgenic mice from SARS-CoV-2 infection.

Thus, we used a combination of Vero E6 cells, Huh-7 cells, and 293T-hACE2 cells, to evaluate the anti-SARS-CoV-2 effects of chloroquine, bafilomycin A1 and NH_4_CL. Moreover, hACE2 transgenic mice were used as an animal model to mimic pathological changes associated with SARS-CoV-2-induced pneumonia. This study demonstrated that these endosomal acidification inhibitors exhibited anti-viral actions against SARS-CoV-2. Future researches are needed to investigate the specific mechanisms underlying these effects.

## Conclusions

In summary, this study demonstrated the anti-SARS-CoV-2 effects of several endosomal acidification inhibitors (chloroquine, bafilomycin A1, and NH_4_CL) in Vero E6, Huh-7 and 293T-hACE2 cells, and in hACE2 transgenic mice. This study provides an experimental basis and supplemental information for targeting endosomal acidification in the development of SARS-CoV-2 therapeutics.

## Data Availability

All data generated or analyzed during this study are included in this published article.

## Abbreviations

ACE2: Angiotensin-converting enzyme 2
MOI: Multiplicity of infection
DMEM: Dulbecco’s modified Eagle medium
FBS: Fetal bovine serum
DEPC: Diethyl phosphorocyanidate
DIG: Digoxin
HRP: Horseradish Peroxidase
DAB: 3,3′-Diaminobenzidine
H&E: Hematoxylin and eosin
SEM: Standard error of mean
ANOVA: Analysis of variance
EC_50_: Concentration for 50% of maximal effect
PFU: Plaque forming units
